# Immunopathologic characteristics of Chinese pediatric patients with chronic rhinosinusitis^☆^

**DOI:** 10.1016/j.waojou.2021.100616

**Published:** 2021-12-05

**Authors:** Lijie Jiang, Yinhui Zeng, Zhaoqi Huang, Yiquan Tang, Qingxiang Zeng, Wenlong Liu, Jianbo Shi

**Affiliations:** a Otorhinolaryngology Hospital, The First Afﬁliated Hospital, Sun Yat-sen University, Guangzhou, 510080, China; b Department of Otolaryngology, Guangzhou Women and Children's Medical Center, Guangzhou Medical University, Guangzhou, China

**Keywords:** Chronic rhinosinusitis with nasal polyps, Chronic rhinosinusitis without nasal polyps, Histopathology, Pediatric, Th1/Th2/Th17 cytokines

## Abstract

**Background:**

The histopathology of pediatric chronic rhinosinusitis with nasal polyps (CRSwNP) and without nasal polyps (CRSsNP) is rarely reported due to low prevalence or the unavailability of tissue samples. Hence, we aimed to characterize and compare the histologic features and protein expression of Th1/Th2/Th17-related cytokines in pediatric CRSsNP and CRSwNP.

**Methods:**

The histologic characteristics of 15 children with CRSsNP, 52 children with CRSwNP, and 12 control participants were analyzed using hematoxylin and eosin staining. The expression of Th1/Th2/Th17-related cytokines were examined using immunohistochemistry and the enzyme-linked immunosorbent assay.

**Results:**

Pediatric subjects with CRSwNP had more intact epithelium and less submucosal mucous glands compared to those with CRSsNP. Tissue eosinophils were more prevalent in the younger CRSwNP group compared to the older CRSwNP or the CRSsNP groups. The protein concentrations of Th2 cytokines were significantly higher in the CRSwNP group than the CRSsNP group or the control group. Moreover, the protein concentrations of Th17 cytokines were significantly higher in the younger CRSwNP group than the older CRSwNP group or the CRSsNP and control groups. The protein concentrations of Th1 and Th17 cytokines were also significantly higher in the CRSsNP group than the control group. Compared with non-eosinophilic CRSwNP, eosinophilic CRSwNP presented with elevated protein concentrations of Th1 and Th17 cytokines.

**Conclusion:**

For the first time, we showed that pediatric CRSwNP presents as eosinophilic with Th2/Th17 inflammation, whereas CRSsNP presents as Th1/Th17 inflammation. Our study may provide a theoretical basis for the precise treatment of pediatric CRS in the future.

## Introduction

Chronic rhinosinusitis (CRS) is common in both the adult and child populations.^1^^,^^2^ CRS is often grouped as chronic rhinosinusitis with nasal polyps (CRSwNP) and without nasal polyps (CRSsNP). In children, nasal polyps (NP) are a rare condition with prevalence of 0.1%.^3^

The histopathology of pediatric CRSsNP and CRSwNP is rarely reported due to low prevalence or the unavailability of tissue samples. Previous research found that eosinophilic tissue infiltration is more obvious in adults and in older children than in younger children with CRS.^4^ The study by Coffinet et al found that lymphocytes were more prevalent in young children with CRS, rather than eosinophils.^5^ For CRSwNP, Mitroi et al. demonstrated that T cells and eosinophils were the main inflammatory cells in NPs; however, their study included both children and adults who have not been grouped according to age.^6^

Moreover, an increasing number of studies had revealed that the immunopathologic features of CRSsNP and CRSwNP are different between various ethnic groups, suggesting that different mechanisms contribute to the pathology of these conditions.^7^^,^^8^ CRSsNP is believed to be a Th1-dominant inflammation, whereas Th2 and Th17 inflammations are observed in eosinophilic rather than in non-eosinophilic CRSwNP.^7^, ^8^, ^9^ For the expression of Th cytokines, Cao et al demonstrated that Th1 responses are dominant in Chinese patients with CRSsNP, whereas Th2 responses are found in eosinophilic CRSwNP rather than all CRSwNP types.^8^ But now Delemarre et al reported that CRSsNP also with a moderate type 2 immune response showed a considerable eosinophilic inflammation with clinical impact.^10^

In this study, we characterized and compared the histologic features and the protein expression of Th1/Th2/Th17-related cytokines in pediatric CRSsNP and CRSwNP.

## Methods

### Patients

This study recruited 15 children with CRSsNP, 52 children with CRSwNP, and 12 children without any sinonasal disease who have undergone optic nerve decompression due to traumatic optic neuropathies (control group) from the medical institution A and B between July 2016 and April 2020. CRSsNP or CRSwNP was determined according to persistent nasal symptoms (>3 months), nasal endoscopic examination, and computed tomographic scans as described by the 2020 European Position Paper on Rhinosinusitis and Nasal Polyps.^11^ All enrolled patients did not respond to maxima medical therapy or adenoidectomy.^12^^,^^13^ Asthma or allergic rhinitis were diagnosed according to lung function and through allergen tests.^14^^,^^15^ All study participants ceased their usage of oral or local corticosteroids and antibiotics 1 month before surgery. Diseased unciform process mucosal samples from patients with CRSsNP, polyp tissues from patients' middle nasal meatus with CRSwNP, and unciform process from control subjects were collected. Patients with prior sinus surgery, immunodeficiency, cystic fibrosis, and Primary ciliary dyskinesia were excluded.^16^, ^17^, ^18^ This study was approved by the local ethical committee ([2016]096) and informed consent was obtained from the children's guardians.

According to“Clinical Consensus Statement: Pediatric Chronic Rhinosinusitis”, the management of children aged 12 years and younger with CRS is distinctly different than management of children aged 13 to 18 years old with CRS，which had raised by Brietzke (Clinical Consensus Statement: Pediatric Chronic Rhinosinusitis). Therefore, we divided the enrolled patients into 6–12 years and 13–18 years groups.

### Morphological study

Paraffin-embedded sections were stained using hematoxylin and eosin. Tissue eosinophilia was confirmed when the mean eosinophil count from five separate counts was >10% under the high power ﬁeld (HPF).^18^, ^19^, ^20^ The blood cell counts were performed using the LH-785 system (Beckman Coulter, Ireland).

Epithelial integrity was defined as the proportion of intact epithelium in the entire sample length (400 × ). The epithelial thickness was obtained by measuring 10 random epithelia in each slide (400 × ). The basement membrane thickness was scored as follows (400 × ): 0 = none (<7.5 μm); 1 = mild (7.5 μm–15 μm); 2 = moderate (15 μm–30 μm); or 3 = marked (>30 μm).^21^, ^22^, ^23^ The submucosa gland hyperplasia in 1 section was scored as follows (100 × ): 0 (<3 glands); 1 (3–10 glands); 2 (11–30 glands); or 3 (>30 cells).^21^

For immunohistochemistry (IHC), the sections were stained overnight at 4 °C using rabbit or mouse antibodies against IFN-γ, IL-4, IL-5, IL-13, IL-17, and IL-23 (Abcam, USA), and then with biotinylated goat anti-mouse/rabbit IgG secondary antibody. Phosphate buffer saline（PBS） was used as the control.^24^

### Enzyme-linked immunosorbent assay (ELISA)

Tissue homogenates were prepared as previously described.^25^ The IFN-γ, IL-4, IL-5, IL-13, IL-17, and IL-23 protein concentrations were assessed using ELISA kits (R&D, USA) according to the manufacturer's instructions.

### Statistical analyses

The data were presented as mean ± SD, except for those with additional notes. Despite the sample size between the rhinosinusitis groups and controls was evident, the difference of sample size among groups was acceptable when the CRSwNP group was divided into 6–12 y (n = 24) and 13–18 y (n = 28) CRSwNP subgroup. The Mann-Whitney *U* test was performed for the comparison of 2 groups, and the Kruskal-Wallis test with Dunn correction was performed in multiple comparisons. Statistical significance was set at *P* < 0.05.

## Results

### Demographic characteristics of the study population

Our study included 52 children with CRSwNP, 15 children with CRSsNP, and 12 children in the control group. Their demographic characteristics are presented in Table 1. No statistically significant difference in demographic characteristics was observed between the CRSsNP and CRSwNP groups. Nevertheless, we found that both CRSsNP and CRSwNP were more prevalent in boys than in girls.

**Table 1:** Demographic characteristics of the study population

	CRSsNP	CRSwNP	Control
Numbers	15	52	12
Girls, n (%)	2 (13.3%)	8 (15.4%)	6 (50%)
Age (y), mean ± SD	13.4 ± 3.8	12.5 ± 3.1	11.5 ± 6.2
Allergic rhinitis, n (%)	2 (13.3%)	6 (11.5%)	0 (0%)
Asthma, n (%)	0 (0%)	1 (1.9%)	0 (0%)

Abbreviations: CRSsNP, chronic rhinosinusitis without nasal polyps; CRSwNP, chronic rhinosinusitis with nasal polyps.

### Histopathology between pediatric CRSsNP and CRSwNP

**Fig. S1:**
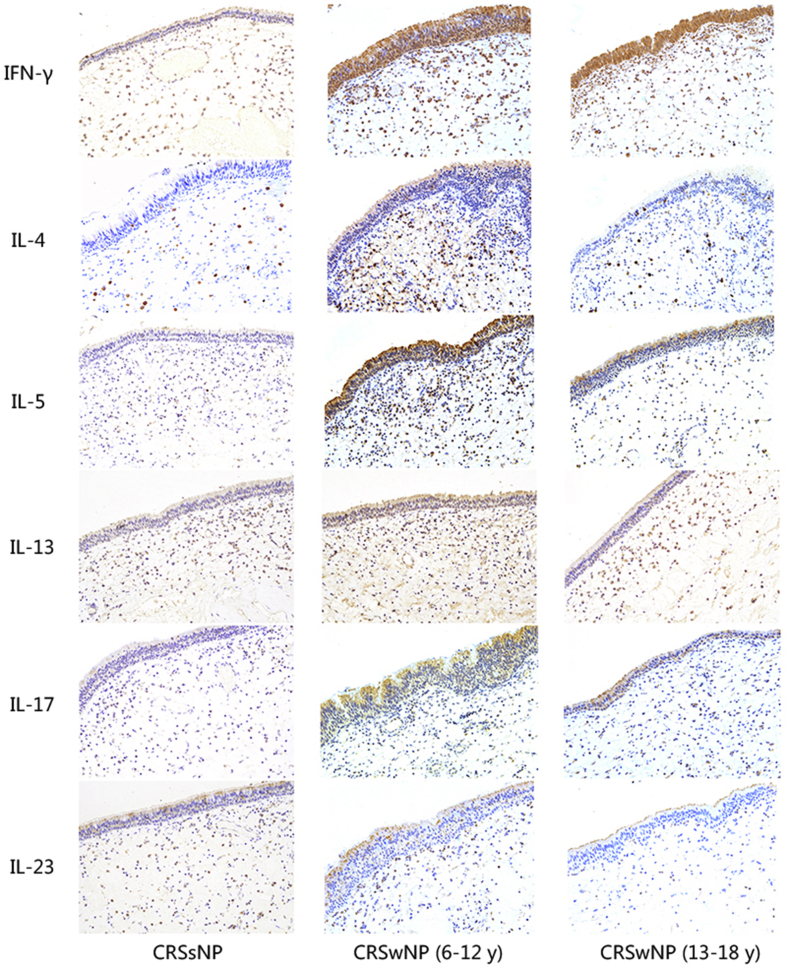
Representative graphs of HE-stained sinus mucosal tissue (original magnification: 400 × ) from pediatric CRSsNP or CRSwNP. (A) CRSsNP, incomplete epithelium and more submucosal mucous glands. (B) CRSsNP, intact epithelium and less submucosal mucous glands. (C-E). The eosinophils in the CRSsNP (C), young CRSwNP (D) and old CRSwNP (E) groups. Abbreviations: CRSsNP, chronic rhinosinusitis without nasal polyps; CRSwNP, chronic rhinosinusitis with nasal polyps; HE, hematoxylin and eosin

Pediatric subjects in 6–12y or 13-18y CRSwNP group had more intact epithelium and less submucosal mucous glands than those with CRSsNP (P < 0.05). However, the thickness of the epithelium and the basement membranes was not different between the CRSsNP and CRSwNP groups (P > 0.05) (Table 2, Fig. S1). The tissue eosinophil proportion was significantly higher in the 6-12y CRSwNP group (48%) than in the 13-18y CRSwNP group (22%) or the CRSsNP group (Table 3, Fig. S1). However, the blood neutrophil, blood eosinophil, and tissue neutrophil counts were not significantly different among the various subgroups (P > 0.05) (Table 3).

**Table 2:** Morphologic characteristics of CRSsNP and CRSwNP as per HE staining

	CRSsNP		CRSwNP
	6-18 y (n = 15)	6-12 y (n = 24)	13-18 y (n = 28)
Epithelial integrity, %	55.38 ± 35.553^a^	72.174 ± 38.93	72.481 ± 35.714
Mucus glands score	1.267 ± 1.335^a^	0.957 ± 1.147	0.963 ± 1.018
Epithelium, μm	24.357 ± 13.001	23.368 ± 15.214	24.8 ± 12.768
Basement membrane, μm	10 ± 3.803	12.079 ± 4.62	11.625 ± 5.878

The data were expressed as mean ± SD.

Abbreviations: CRSsNP, chronic rhinosinusitis without nasal polyps; CRSwNP, chronic rhinosinusitis with nasal polyps; HE, hematoxylin and eosin.

a Compared with 6–12 y or 13–18 y CRSwNP group, *P* < 0.05.

**Table 3:** The submucosal inflammatory cell of CRSsNP and CRSwNP by HE staining

	CRSsNP		CRSwNP			
	6-18 y (n = 15)		6-12 y (n = 24)		13-18 y (n = 28）	
	Absolute value	proportion	Absolute value	proportion	Absolute value	proportion
Blood NEU	4.138 ± 1.22	0.536 ± 0.134	3.707 ± 1.869	0.467 ± 0.129	4.971 ± 2.17	0.62 ± 0.121
Blood EOS	0.046 ± 0.05	0.006 ± 0.005	0.041 ± 0.026	0.005 ± 0.003	0.027 ± 0.021	0.004 ± 0.003
Tissue EOS	11.514 ± 15.279	0.093 ± 0.135	14.93 ± 18.087	0.131 ± 0.162^a^	12.359 ± 18.402	0.095 ± 0.137
Tissue NEU	1.421 ± 2.192	0.011 ± 0.018	1.373 ± 1.952	0.013 ± 0.018	1.642 ± 2.352	0.012 ± 0.018

The data were expressed as mean ± SD.

Abbreviations: NEU, neutrophils; EOS, eosinophils; CRSsNP, chronic rhinosinusitis without nasal polyps; CRSwNP, chronic rhinosinusitis with nasal polyps; HE, hematoxylin and eosin.

a Compared with CRSsNP or 13–18 y CRSwNP, *P* < 0.05.

### The expression of Th1/Th2/Th17-related cytokines in pediatric CRSsNP and CRSwNP

**Fig. S2:**
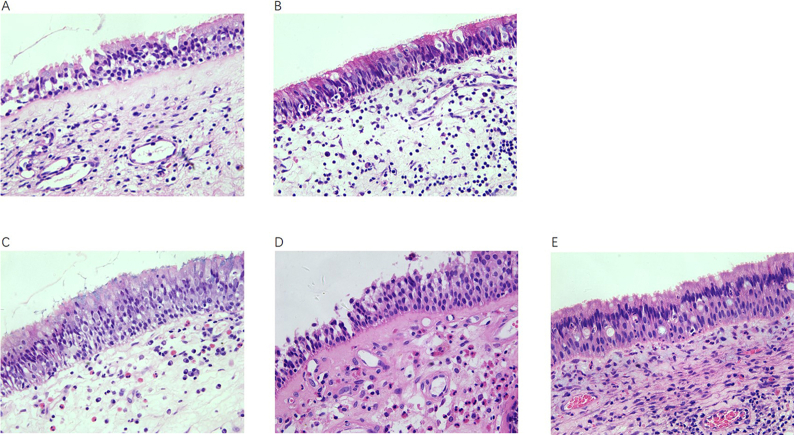
Representative graphs of immunohistochemistry for Th1/Th2/Th17 cytokine staining (original magnification: 200 × ) in the CRSsNP, young CRSwNP, and old CRSwNP groups. Abbreviations: CRSsNP, chronic rhinosinusitis without nasal polyps; CRSwNP, chronic rhinosinusitis with nasal polyps

The IFN-γ positive cell counts were not significantly different among the various subgroups (*P* > 0.05) (Table 4, Fig. S2). By contrast, the IL-5, IL-13, IL-17, and IL-23 positive cell counts in the 6-12y CRSwNP group were significantly higher compared with the 13-18y CRSwNP or the CRSsNP groups (*P* < 0.05) (Table 4, Fig. S2). Moreover, the IL-4 positive cell count was significantly higher in the 6-12y and 13-18y CRSwNP groups compared to the CRSsNP group (*P* < 0.05) (Table 4, Fig. S2).

**Table 4:** The expression of Th1/Th2/Th17-related cytokines between pediatric CRSsNP and CRSwNP as per immunochemistry

	CRSsNP	CRSwNP	
	6-18 y (n = 15)	6-12 y (n = 24)	13-18 y (n = 28)
IFN-γ	0.522 ± 0.294	0.56 ± 0.331	0.504 ± 0.307
IL-4	0.023 ± 0.011	0.068 ± 0.104^b^	0.044 ± 0.039^b^
IL-5	0.165 ± 0.104	0.228 ± 0.195^a^	0.131 ± 0.083
IL-13	0.265 ± 0.217	0.433 ± 0.215^a^	0.321 ± 0.209
IL-17	0.182 ± 0.15	0.251 ± 0.166^a^	0.116 ± 0.079
IL-23	0.138 ± 0.074	0.203 ± 0.107^a^	0.15 ± 0.08

The data were expressed as mean ± SD.

Abbreviations: CRSsNP, chronic rhinosinusitis without nasal polyps; CRSwNP, chronic rhinosinusitis with nasal polyps.

a Compared with CRSsNP or 13–18 y CRSwNP, *P* < 0.05.

b Compared with CRSsNP, *P* < 0.05.

### Protein expression of Th1/Th2/Th17-related cytokines by tissue

The IFN-γ protein concentrations were significantly elevated in patients with CRSsNP, as well as among the 6-12y or 13-18y CRSwNP groups, compared to the 6-12y or 13-18y control group (*P* < 0.05) (Table 5). The protein concentrations of Th2 cytokines (IL-4, IL-5, IL-13) were significantly higher in both the 6-12y or 13-18y CRSwNP groups compared to the CRSsNP and the 6-12y or 13-18y control groups (*P* < 0.05) (Table 5). The protein concentrations of Th17 cytokines (IL-17, IL-23) were significantly higher in the 6-12y CRSwNP group compared to the13-18y CRSwNP and CRSsNP groups and the 6-12y or 13-18y control group (*P* < 0.05) (Table 5). The protein concentrations of Th1 and Th17 cytokines were significantly higher in the CRSsNP group compared to the 6-12y or 13-18y control group (*P* < 0.05) (Table 5).

**Table 5:** The tissue protein expression of Th1/Th2/Th17-related cytokines as per ELISA

	CRSsNP	CRSwNP		Controls	
	6-18 y (n = 15)	6-12y (n = 24)	13-18 y (n = 28)	6-12 y (n = 5)	13-18 y (n = 7)
IFN-γ	39.5 ± 21.7^b^	46.8 ± 31.2^b^	42.3 ± 22.6^b^	19.4 ± 15.3	23.6 ± 17.8
IL-4	–	59.7 ± 18.5	54.6 ± 12.8	–	–
IL-5	18.6 ± 9.6	183.2 ± 65.6^a^^b^	133.9 ± 58.5^a^^b^	12.5 ± 6.6	14.8 ± 9.2
IL-13	168.9 ± 41.5	278.6 ± 76.3^a^	213.4 ± 48.7^a^	–	–
IL-17	239.5 ± 173.6^b^	573.2 ± 288.1^a^^b^	146.8 ± 101.9^b^	41.5 ± 13.7	49.8 ± 16.3
IL-23	173.6 ± 99.5	378.2 ± 119.6^a^	211.3 ± 108.5	–	–

The data were expressed as mean ± SD.

The protein concentrations are in pg/mL.

Abbreviations: CRSsNP, chronic rhinosinusitis without nasal polyps; CRSwNP, chronic rhinosinusitis with nasal polyps; ELISA, enzyme-linked immunosorbent assay.

a Compared with CRSsNP or 13–18 y CRSwNP, *P* < 0.05.

b Compared with control, *P* < 0.05.

### Comparison of cytokine expression between eosinophilic and non-eosinophilic CRSwNP and CRSsNP

Eosinophilic and non-eosinophilic CRSsNP account for 40% and 60% of the CRSsNP group, respectively. By contrast, eosinophilic and non-eosinophilic CRSwNP account for 36.5% and 63.5% of the CRSwNP, respectively.

Compared to non-eosinophilic CRSsNP, eosinophilic CRSsNP was found to have increased protein concentrations of IFN-γ, IL-5, IL-13, and IL-17 (*P* < 0.05) (Table 6). Similarly, compared to non-eosinophilic CRSwNP, eosinophilic CRSwNP had increased protein concentrations of IFN-γ, IL-5, IL-17, and IL-23 (*P* < 0.05) (Table 6).

**Table 6:** The expression of Th1/Th2/Th17-related cytokines between pediatric CRSsNP and CRSwNP as per immunochemistry

Cytokines	CRSsNP		CRSwNP	
	EOS(n = 6)	Non-EOS(n = 9)	EOS(n = 18)	Non-EOS (n = 34)
EOS	0.223 ± 0.125^a^	0.012 ± 0.015	0.278 ± 0.16^a^	0.032 ± 0.026
IFN-γ	0.694 ± 0.179^a^	0.427 ± 0.31	0.675 ± 0.325^a^	0.483 ± 0.287
IL-4	0.021 ± 0.013	0.025 ± 0.01	0.051 ± 0.053	0.059 ± 0.089
IL-5	0.235 ± 0.096^a^	0.119 ± 0.085	0.256 ± 0.199^a^	0.132 ± 0.086
IL-13	0.45 ± 0.218^a^	0.141 ± 0.101	0.41 ± 0.216	0.369 ± 0.221
IL-17	0.261 ± 0.186^a^	0.122 ± 0.088	0.242 ± 0.186^a^	0.151 ± 0.11
IL-23	0.162 ± 0.085	0.121 ± 0.066	0.212 ± 0.102^a^	0.154 ± 0.089

The data were expressed as mean ± SD.

Abbreviations: EOS, eosinophils; CRSsNP, chronic rhinosinusitis without nasal polyps; CRSwNP, chronic rhinosinusitis with nasal polyps.

a Compared with Non-EOS, *P* < 0.05.

## Discussion

In the current study, we investigated and compared the pathological patterns and concentrations of Th-related cytokines in CRSsNP and CRSwNP among Chinese children. To the best of our knowledge, this is the first time that the immunologic endotypes of CRS was explored among Chinese children.

We found that pediatric subjects with CRSwNP had more intact epithelium and less submucosal mucous glands compared to those with CRSsNP. Similarly, Chan et al. reported that pediatric CRS is characterized by thinner epithelium and basement membranes, and less submucosal mucous glands.^4^

Eosinophilic and neutrophilic inflammation are the two main CRS inflammatory types in both children and adults.^26^^,^^27^ Berger et al and Coffinet et al showed that tissue eosinophilia is significantly greater in adult CRS compared to pediatric CRS.^5^^,^^28^ However, Baroody et al found that eosinophilic inflammation was prevalent in children with refractory CRS.^29^ Our present study suggests that the CRS pathological type in Chinese children is significantly different from those of Caucasian children. Moreover, eosinophilic inflammation is more prevalent in the young CRSwNP group (48%) than the old CRSwNP group (22%) in our study, which is opposite from the inflammation pattern observed among Caucasian children.^4^ Moreover, we found that non-eosinophilic infiltration accounts for most inflammation patterns (>60%) among children with CRSwNP or CRSsNP. This is consistent with the results of a study by Cao et al, which involved Chinese adults with CRS.^8^ However, the neutrophilic inflammation in all types of pediatric CRS is very minor compared to that in adult CRS.^13^ To exclude the problem of antibodies, we tested 2 types of antibodies from different suppliers (RD BAF3174 and abcam9535) a, and arrived at similar results. These differences between Chinese adult and pediatric populations with CRS may be attributed to genetics and environmental factors.

An increased Th2/Th17 response was found in eosinophilic CRSwNP rather than in non-eosinophilic CRSwNP. Wang et al. found that adult CRSwNP in Beijing presented mixed patterns of Th1/Th2/Th17 expression, whereas subjects from Chengdu present with a lower Th2 expression.^25^ Interestingly, Baba et al. found elevated Th2 cytokine levels in eosinophilic CRS without Th17 cytokines in the Japanese population.^30^ Our previous study also found that an eosinophilic shift of diffuse rhinosinusitis inflammatory pattern in southern China over the last 18 years.^24^ These pathological differences among similar populations could possibly be attributed to environmental factors.

Our qualitative (IHC) and quantitative (ELISA) analyses suggest that pediatric CRSwNP (regardless of age) presented as Th1/Th2 inflammation, whereas CRSsNP presented as Th1/Th17 inflammation. Moreover, Th1/Th2/Th17 inflammation was more prevalent in the young CRSwNP group than the old CRSwNP group, which may be explained by the mutual enhancement of Th2 and Th17 inflammation. Moreover, the high Th2 and Th17 cytokine levels in the young CRSwNP group may be also explained by the higher eosinophilic inflammation in this group. Consistently, when the subjects were grouped according to the proportion of eosinophils, we also found that both eosinophilic CRS and CRSwNP presented as Th2/Th17 inflammation.

There are several limitations to this study. First, the small sample size used in this study remains as a limitation towards conducting further analysis. Further multi-center studies and larger sample that compare CRS are therefore needed. Second, we did not perform IHC in the control group due to the limited availability of samples. Third, the effect of Th cytokines on dispersed nasal polyp cells was not explored in this study.

In conclusion, this study showed for the first time that pediatric CRSwNP presents as eosinophilic with Th2/Th17 inflammation, whereas CRSsNP presents as Th1/Th17 inflammation. With age, an increasing number of pediatric CRSwNP cases present as non-eosinophilic inflammation, which is similar to the inflammation pattern in adult CRS. Our study may provide a theoretical basis for the precise treatment of pediatric CRS in the future.

## Abbreviations

EOS, Eosinophils; NEU, Neutrophils; CRSsNP, Chronic rhinosinusitis without nasal polyps; CRSwNP, Chronic rhinosinusitis with nasal polyps; ELISA, Enzyme-linked immunosorbent assay; HE, Hematoxylin and eosin.

## Declaration

All the authors consent to the publication of this manuscript.

## Ethical statement

The study approved by the Ethics Committee of the First Affiliated Hospital of Sun Yat-sen University ([2016]096) and informed consent was obtained from the children's guardians.

## Consent for publication

Not applicable.

## Data availability statement

All data generated or analyzed during this study are included in this published article and its supplementary information files.

## Funding

This study was funded by grants from the National Natural Science Grant of China (No. 81970861), the Guangdong Province Natural Science Grant (No. 2021A1515010940), and the Science and Technology Program of Guangzhou (No. 202102020079).

## Authors' contributions

Study design: Wenlong Liu, Jianbo Shi; experiment: Lijie Jiang, Yinhui Zeng, Zhaoqi Huang; data collected and analysis: Lijie Jiang, Yinhui Zeng, Zhaoqi Huang, Qingxiang Zeng; manuscirpt drafting: Wenlong Liu and Lijie Jiang.

## Declaration of competing interest

The authors declare that they have no relevant conflicts of interest.

## References

[1] Bachert C., Marple B., Schlosser R.J. (2020). Adult chronic rhinosinusitis. Nat Rev Dis Primers.

[2] Rose A.S., Thorp B.D., Zanation A.M., Ebert C.S. (2013). Chronic rhinosinusitis in children. Pediatr Clin.

[3] Settipane G.A. (1996). Epidemiology of nasal polyps. Allergy Asthma Proc.

[4] Chan K.H., Abzug M.J., Coffinet L., Simoes E.A.F., Cool C., Liu A.H. (2004). Chronic rhinosinusitis in young children differs from adults: a histopathology study. J Pediatr.

[5] Coffinet L., Chan K.H., Abzug M.J., Simões E.A.F., Cool C., Liu A.H. (2009). Immunopathology of chronic rhinosinusitis in young children. J Pediatr.

[6] Mitroi M., Albulescu D., Capitanescu A. (2019). Differences in the distribution of CD20, CD3, CD34 and CD45RO in nasal mucosa and polyps from patients with chronic rhinosinusitis. Mol Med Rep.

[7] Zhang N., Van Zele T., Perez-Novo C. (2008). Different types of T-effector cells orchestrate mucosal inflammation in chronic sinus disease. J Allergy Clin Immunol.

[8] Cao P.-P., Li H.-B., Wang B.-F. (2009). Distinct immunopathologic characteristics of various types of chronic rhinosinusitis in adult Chinese. J Allergy Clin Immunol.

[9] Cho S.H., Hamilos D.L., Han D.H., Laidlaw T.M. (2020). Phenotypes of chronic rhinosinusitis. J Allergy Clin Immunol Pract.

[10] Delemarre T., Holtappels G., De Ruyck N. (2020). Type 2 inflammation in chronic rhinosinusitis without nasal polyps: another relevant endotype. J Allergy Clin Immunol.

[11] Fokkens W.J., Lund V.J., Hopkins C. (2020). European position paper on rhinosinusitis and nasal polyps 2020. Rhinology.

[12] Chandy Z., Ference E., Lee J.T. (2019). Clinical guidelines on chronic rhinosinusitis in children. Curr Allergy Asthma Rep.

[13] Snidvongs K., Sangubol M., Poachanukoon O. (2020). Pediatric versus adult chronic rhinosinusitis. Curr Allergy Asthma Rep.

[14] Cheng L., Chen J., Fu Q. (2018). Chinese society of allergy guidelines for diagnosis and treatment of allergic rhinitis. Allergy Asthma Immunol Res.

[15] Boulet L.-P., Reddel H.K., Bateman E., Pedersen S., FitzGerald J.M., O'Byrne P.M. (2019). The global initiative for asthma (GINA): 25yearslater. Eur Respir J.

[16] Lahiri T., Hempstead S.E., Brady C. (2016). Clinical practice guidelines from the cystic fibrosis foundation for preschoolers with cystic fibrosis. Pediatrics.

[17] Pereira R., Barbosa T., Gales L. (2019). Clinical and genetic analysis of children with kartagener syndrome. Cells.

[18] Conley M.E., Notarangelo L.D., Etzioni A. (1999). Diagnostic criteria for primary immunodeficiencies. Representing PAGID (pan-American group for immunodeficiency) and ESID (European society for immunodeficiencies). Clin Immunol.

[19] Wang K., Deng J., Yang M. (2019). Concordant systemic and local eosinophilia relates to poorer disease control in patients with nasal polyps. World Allergy Organ J.

[20] Lou H., Zhang N., Bachert C., Zhang L. (2018). Highlights of eosinophilic chronic rhinosinusitis with nasal polyps in definition, prognosis, and advancement. Int Forum Allergy Rhinol.

[21] Dhong H.-J., Kim H.Y., Cho D.-Y. (2005). Histopathologic characteristics of chronic sinusitis with bronchial asthma. Acta Otolaryngol.

[22] Do T.Q., Barham H.P., Earls P. (2016). Clinical implications of mucosal remodeling from chronic rhinosinusitis. Int Forum Allergy Rhinol.

[23] Kule Z.G., Habesoglu T.E., Somay A., Deveci H.S., Kule M., Gursel A.O. (2014). Histopathological characteristics of nasal polyps in smokers and non-smokers. J Craniofac Surg.

[24] Luo X., Xu Z., Zuo K. (2021). The changes of clinical and histological characteristics of chronic rhinosinusitis in 18 years: Was there an inflammatory pattern shift in southern China?. World Allergy Organ J.

[25] Wang X., Zhang N., Bo M. (2016). Diversity of T cytokine profiles in patients with chronic rhinosinusitis: a multicenter study in Europe, Asia, and Oceania. J Allergy Clin Immunol.

[26] Bachert C., Akdis C.A. (2016). Phenotypes and emerging endotypes of chronic rhinosinusitis. J Allergy Clin Immunol Pract.

[27] Heath J., Hartzell L., Putt C., Kennedy J.L. (2018). Chronic rhinosinusitis in children: pathophysiology, evaluation, and medical management. Curr Allergy Asthma Rep.

[28] Berger G., Kogan T., Paker M., Berger-Achituv S., Ebner Y. (2011). Pediatric chronic rhinosinusitis histopathology: differences and similarities with the adult form. Otolaryngol Head Neck Surg.

[29] Baroody F.M., Hughes C.A., McDowell P., Hruban R., Zinreich S.J., Naclerio R.M. (1995). Eosinophilia in chronic childhood sinusitis. Arch Otolaryngol Head Neck Surg.

[30] Baba S., Kagoya R., Kondo K., Suzukawa M., Ohta K., Yamasoba T. (2015). T-cell phenotypes in chronic rhinosinusitis with nasal polyps in Japanese patients. Allergy Asthma Clin Immunol.

